# Changes in the Prevalence of Tobacco Consumption and the Profile of Spanish Smokers after a Comprehensive Smoke-Free Policy

**DOI:** 10.1371/journal.pone.0128305

**Published:** 2015-06-11

**Authors:** Monica Perez-Rios, Esteve Fernandez, Anna Schiaffino, Manel Nebot, Maria Jose Lopez

**Affiliations:** 1 Epidemiology Unit, Galician Directorate for Public Health, Galician Health Authority, Xunta de Galicia, Santiago de Compostela, Spain; 2 Biomedical Research Centre Network for Epidemiology and Public Health (CIBERESP), Granada, Spain; 3 Department of Preventive Medicine and Public Health, School of Medicine, University of Santiago de Compostela, Santiago de Compostela, Spain; 4 Tobacco Control Unit, Cancer Control and Prevention Programme, Institut Català d’Oncologia (ICO), Institutd’InvestigacióBiomèdica de Bellvitge (IDIBELL), L’Hospitalet de Llobregat, Barcelona, Spain; 5 Department of Clinical Sciences, School of Medicine, Universitat de Barcelona, L'Hospitalet del Llobregat, Barcelona, Spain; 6 Agència de Salut Pública de Barcelona, Barcelona, Spain; 7 Institute of Biomedical Research (IIB Sant Pau), Barcelona, Spain; Queensland University of Technology, AUSTRALIA

## Abstract

**Background:**

A partial smoke-free regulation in Spain was introduced on January 1, 2006, which was subsequently amended to introduce a comprehensive smoke-free policy from 2 January 2011 onward. The objective of this study was to compare the prevalence of tobacco consumption in Spain and the profile of smokers before (2006) and after (2011) the comprehensive smoking ban passed in 2010.

**Methods:**

Two independent, cross-sectional, population-based surveys were carried out among the adult (≥ 18 years old) Spanish population in 2006 and 2011 through telephone interviews. Both surveys used the same methods and questionnaire. Nicotine dependence was assessed with the Fagerström Test for nicotine dependence and readiness to quit according to the stages of change.

**Results:**

The prevalence of tobacco consumption showed a nonsignificant decrease from 23.4% in 2006 to 20.7% in 2011. No changes were observed in nicotine dependence or readiness to quit. In 2011, most smokers (76%) showed low nicotine dependence and were mainly in the precontemplation stage (72%).

**Conclusions:**

The prevalence of smokers has slightly decreased since the introduction of the total smoking ban in Spain. No differences were found in nicotine dependence or readiness to quit.

## Introduction

A partial smoking ban-a law of health measures against smoking, and the regulation of the sale, supply, consumption, and advertising of tobacco products- was implemented on January 1, 2006 in Spain, one of the countries with the most lax and oldest smoking legislation in Europe at that time. This smoke-free law was highly controversial and generated strong debate, mainly because of the partial ban in hospitality venues. The law banned smoking in all indoor public places and workplaces, but tobacco consumption continued to be allowed in bars and restaurants of less than 100 m² and hospitality venues over 100 m² could designate a physically separate smoking area (occupying 30% of the total area of the venue). Finally, in December 2010, the Spanish Parliament passed a comprehensive smoking law amending and strengthening the previous ban. The amended law extended smoking restrictions to all hospitality premises, thereby making Spanish workplaces smoke-free from January 2, 2011. Previous studies concluded that, in communities where workplaces are smoke-free, smokers are more likely to quit.In european countries where smoke-free laws have been evaluated, the impact of smoke-free legislation has been positive and has prompted a decrese in the prevalence of tobacco consumption [1, 2]. To the best of our knowledge, no studies have evaluated this issue in Spain in a representative population sample of adults.

In 2006, an ad hoc cross-sectional study [3, 4] provided relevant data to characterize tobacco consumption and exposure to second-hand smoke at the national level. That study set the prevalence of smokers at 23.4% (26.7% in men and 21.1% in women), and characterized smokers as having low dependence and being mainly in the precontemplation stage. To assess the possible impact of the comprehensive legislation on smoking, that cross-sectional study was repeated in 2011 in the present study. The aims of this study were to assess the prevalence of smoking among the Spanish general population before (2006) and after (2011) the comprehensive smoking ban came into force and to assess possible changes in the profile of smokers.

## Methods

In 2006 (during June and July) and 2011 (from September to November), two independent cross-sectional surveys assessing specific factors related to tobacco consumption and exposure to second-hand smoke were carried out in a representative sample of the non-institutionalized Spanish population aged 18 years and older. The two studies had similar designs, which have been reported elsewhere [3]. Briefly, a computer-assisted telephone interview was conducted in both studies by applying the same core questions. The study participants were selected by means of a two-stage sampling strategy with stratification in the first order units, i.e. households. To guarantee national representativeness, households were stratified by geographical region and the size of the municipality. Second stage units were residents in the previously selected households, where only one person was selected at random [5]. Households within each municipality were randomly selected using a landline telephone directory as the sampling frame.

The sample size for each survey was calculated to be 2500 people, with similar allocation by sex and age group (18–39 years, 40–59 years, and 60 years and older) to the Spanish population. A total of 2,522 adults were interviewed in 2006 and 2.504 in 2011.

A current smoker was defined as a person who was smoking, whether daily or not, at the time of the survey (591 in 2006 and 518 in 2011). A former smoker was defined as a person who had smoked but was not smoking at the time of the survey, and a non-smoker as a person who reported never smoking. Among cigarette smokers, nicotine dependence was assessed by the Fagerström Test for Nicotine Dependence (FTND) [6], and readiness or intention to quit smoking was ascertained using the Processes of Change Questionnaire, as proposed by Prochaska and DiClemente [7]. Nicotine dependence according to the FTND score was categorized as follows: 0–4 no or low dependence; 5: medium dependence, and 6–10: high nicotine dependence. Intention to quit was classified in three stages: precontemplation, contemplation, and preparation. Precontemplation included those smokers who were not considering quitting; contemplation included those seriously considering quitting within the 6 months following the interview, and preparation comprised those smokers planning to quit within the 30 days following the interview and who had attempted to quit in the past year. This analysis was restricted to cigarette smokers (569 in 2006 and 430 in 2011).

### Statistical analysis

The prevalence of tobacco consumption, nicotine dependence, and stages of change, with 95% confidence intervals (95%CI), was estimated for 2006 and 2011, overall and by sex, age group, and level of education (primary or lower, secondary, and university). Nicotine dependence and stages of change were also calculated by the number of cigarettes smoked per day (<10 cigarettes, 10–19 cigarettes and 20 cigarettes and over). Pearson’s chi-square test for independent samples was used to compare proportions on independent samples. The analysis was performed with Stata v12 on an anonymized dataset.

## Results

As shown in Table 1, the overall prevalence of smokers in 2006 was 23.4% and 20.7% in 2011(p = 0.279). By age, significant changes were observed in the youngest group only: the prevalence of smokers decreased from 30.7% in 2006 to 24.7% in 2011 and the prevalence of never smokers increased from 47.6% to 56.5%, respectively. In 2006 and 2011, the prevalence of smokers was higher among males (p<0.05) and among people with secondary education (p<0.05). The prevalence of smokers rolling tobacco rose from 1.5% in 2006 to 15.6% in 2011, while the percentage of smokers who reported that they smoked manufactured cigarettes declined from 96.3% to 83%. The prevalence of persons smoking 20 or more cigarettes decreased from 31.7% in 2006 to 14.5% in 2011.

**Table 1:** Prevalence of tobacco consumption overall and by sex, age group and level of education (Spain, 2006–2011).

			2006			2011		
		n	%	IC(95%)	n	%	IC(95%)	p
Smokers	Overall	591	23.4	21.8―25.1	518	20.7	19.1―22.3	0.279
	Sex							
	Male	330	27.0	24.5―29.5	279	23.6	21.2―26.0	0.337
	Female	261	20.1	17.9―22.2	239	18.1	16.0―20.2	0.570
	Age group (years)							
	18–39	330	30.7	27.9―33.5	200	24.7	21.7―27.7	0.138
	40–59	215	27.7	24.5―30.8	246	26.1	23.3―29.0	0.699
	60 and older	46	6.9	4.9―8.8	68	9.3	7.2―11.4	0.649
	Level of education							
	Primary or less	207	19.8	17.4―22.2	121	15.5	13,0―18.1	0.330
	Secondary	237	29.3	26.1―32.4	235	26.4	23,5―29.3	0.482
	University	126	22.2	18.8―25.6	160	19.7	16,9―22.4	0.605
Former smokers	Overall	689	27.3	25.6―29.1	767	30.6	28.8―32.4	0.166
	Sex							
	Male	442	36.2	33.5―38.9	449	38.0	35.2―40.8	0.578
	Female	247	19.0	16.9―21.1	318	24.1	21.7―26.4	0.146
	Age group (years)							
	18–39	233	21.7	19.2―24.1	152	18.8	16.1―21.5	0.491
	40–59	251	32.3	29.0―35.6	345	36.7	33.6―39.7	0.257
	60 and older	205	30.6	27.1―34.1	263	35.9	32.4―39.4	0.228
	Level of education							
	Primary or less	258	24.7	22.1―27.3	249	31.9	28.6―35.2	0.013
	Secondary	241	29.8	26.6―32.9	259	29.1	26.1―32.1	0.864
	University	176	31.0	27.2―34.9	252	31.0	27.8―34.2	1
Never smokers	Overall	1242	49.2	47.3―51.2	1219	48.7	46.7―50.6	0.804
	Sex							
	Male	449	36.8	34.1―39.5	454	38.4	35.6―41.2	0.620
	Female	793	61.0	58.3―63.6	765	57.9	55.2―60.5	0.213
	Age group (years)							
	18–39	512	47.6	44.6―50.6	458	56.5	53.1―60.0	0.006
	40–59	311	40.0	36.6―43.5	350	37.2	34.1―40.3	0.460
	60 and older	419	62.5	58.9―66.2	401	54.8	51.2―58.4	0.025
	Level of education							
	Primary or less	581	55.5	52.5―58.6	410	52.6	49.1―56.1	0.367
	Secondary	332	41.0	37.6―44.4	395	44.4	41.2―47.7	0.356
	University	265	46.7	42.6―50.8	401	49.3	45.9―52.8	1

Table 2 shows the distribution of smokers according to nicotine dependence. Notably, between 2006 and 2011, dependence barely changed among smokers. In 2011, 13.7% of smokers had high nicotine dependence. Among smokers of 10 or more cigarettes per day, nicotine dependence increased from 2006 to 2011 (p<0.05). No significant differences were found in the prevalence of smokers who reported they smoked the first cigarette within 30 minutes of getting up.

**Table 2:** Difference between 2006 and 2011 in nicotine dependence among cigarette smokers overall and by sex, age group, educational level and number of cigarettes smoked per day.

		2006				2011		
	n	Low or none^1^ (%)	Medium^2^ (%)	High^3^ (%)	n	Low or none^1^ (%)	Medium^2^ (%)	High^3^ (%)
Overall	569	74.0	10.4	15.6	430	76.0	10.2	13.7
Sex								
Male	310	73.2	10.3	16.5	216	70.4	13.0	16.7
Female	259	74.9	10.4	14.7	214	81.8	7.5	10.7
Age group (years)								
18–39	325	79.4	8.3	12.3	162	76.5	11.1	12.3
40–59	207	65.7	13.5	20.8	212	75.0	9.4	15.6
60 and older	37	73.0	10.8	16.2	53	81.1	9.4	9.4
Level of education								
Primary and less	196	64.8	12.2	23.0	97	71.1	9.3	19.6
Secondary	234	78.6	10.7	10.7	196	75.5	11.7	12.8
University	119	77.3	8.4	14.3	136	80.1	8.8	11.0
Number of cigarettes/day								
< 10	183	99.5	0.5	0.0	212	96.7	3.3	0.0
10–19 *	205	82.9	10.7	6.3	154	69.5	13.6	16.9
> = 20*	180	38.3	20.0	41.7	62	21.0	25.8	53.2

* Differences between 2006 and 2011 p<0.05

^1^Fagerström test score from 0 to 4

^2^Fagerström test score 5

^3^Fagerström test score from 6 to 10

According to Prochaska and DiClemente’s Stages of Change model (Table 3), the percentage of smokers in the preparation stage decreased between 2006 (10.4%) and 2011 (5%) (*p* = 0.005) while that in the precontemplation stage increased (64.3% in 2006 and 72.3% in 2011; *p*<0.05).

**Table 3:** Difference between 2006 and 2011 in stages of change among cigarette smokers overall and by sex, age group, education level and number of cigarettes smoked per day.

		2006				2011		
	n	Precontemplation (%)	Contemplation (%)	Preparation (%)	n	Precontemplation (%)	Contemplation (%)	Preparation (%)
Overall*	569	64.3	25.3	10.4	379	72.3	22.7	5.0
Sex								
Male	310	66.1	22.6	11.3	191	71.7	21.5	6.8
Female*	259	62.2	28.6	9.3	188	72.9	23.9	3.2
Age group (years)								
18–39*	325	65.2	24.3	10.5	141	68.1	27.0	5.0
40–59*	207	63.3	28.0	8.7	189	74.1	21.7	4.2
60 and older	37	62.2	18.9	18.9	48	79.2	14.6	6.3
Level of education								
Primary and less	196	65.3	26.0	8.7	85	74.1	21.2	4.7
Secondary*	234	62.4	25.2	12.4	172	75.6	20.9	3.5
University	119	64.7	24.4	10.9	121	66.1	26.4	7.4
Number of cigarettes/day								
< 10	183	65.0	23.5	11.5	188	72.9	21.3	5.9
10–19	205	61.5	29.3	9.3	132	68.9	26.5	4.5
> = 20	180	66.7	22.8	10.6	58	77.6	19.0	3.4

* Differences between 2006 and 2011 (*p*<0.05)

## Discussion

This study reaffirms the high prevalence of smokers in Spain, where 21% of the adult population smokes. This percentage represents a slight, non-significant decrease compared with the estimated prevalence in 2006. Although nicotine dependence is low, most smokers are still in the precontemplation stage.

The prevalence of smokers significantly decreased in the youngest population group but the data did not allow us to establish a causal relationship between the implementation of the comprehensive smoke-free legislation and changes in the prevalence of smoking, since recent changes reflect the previously observed downward trends [8,9]. Furthermore, there is scarce evidence about the impact of the smoking laws on the prevalence of smoking [10]. Our results are in line with those of a similar study carried out in Spain in the general population in a similar period [11], but a slight decrease in prevalence was observed among Spanish workers [12]. The increase in the prevalence of tobacco consumption in the oldest groups is not a coincidence and reflects changes occurring in tobacco consumption in Spain, where women were mainly non-smokers until the 1980s. When the analysis of smoking prevalence was restricted to the oldest population (75 years and over), the prevalence remained stable (66.8% of non-smokers in 2006 vs. 64.7% in 2011).

In addition to the smoke-free policies, two other meaningful measures of tobacco control were introduced in Spain during the study period: a tax increase on some tobacco products [13], mainly fine-cut tobacco, and pictorial health warnings [14]. These measures could have increased the tobacco control pressure in Spain and could thus have contributed to the slight decrease observed in the prevalence of smoking.

Contrary to the “hardening hypothesis”(the lower the prevalence, the higher the dependence) [15], our findings show that nicotine dependence remained stable. However, this hypothesis was proposed solely on the basis of clinical observations and ecological data [15] and is not supported by a range of studies [16–19].

Our study found an increase in smokers in the precontemplation stage and a decrease in those in the preparation phase, possibly indicating that the number of smokers ready to quit is declining. This would indicate that, although nicotine dependence is decreasing among Spanish smokers, the proportion considering quitting in the short term has also declined. This finding contradicts the results of a study that examined the motivation to quit in 5 countries according to their tobacco control activity [20], which found that the proportion of people intending to quit was higher in countries with a high level of tobacco control activity. However, methodological differences among studies must be taken into consideration.

The main strengths of this study are its large sample size and its population representativeness. It is also noteworthy that the data for 2006 and 2011 analyzed in this study were obtained by applying the same questions. Furthermore, to the best of our knowledge, this is the first study that allows estimation of the impact of the Spanish 2011 smoke-free legislation on the prevalence and characteristics of smokers.

Few studies have considered nicotine dependence and stages of change at the population level, since these characteristics of smoking are mostly considered in the clinical setting and such data is rarely collected in population-based surveys. Nevertheless, some studies have reported that a high proportion of smokers describe themselves as low dependent and at the precontemplative stage [21, 22] in Spain. On a population level, nicotine dependence and stages of change were estimated in the IBERPOC study [23]. This study was conducted in seven areas of Spain in 2006–2007 in a sample of individuals aged 40 to 69 years old to obtain the prevalence of chronic obstructive pulmonary disease and its relationship with smoking. Although the results of the IBERPOC study could not be extrapolated to the general population due to the selection process, 39% of the smokers were classified as precontemplators and 58% as contemplators, these percentages differing from those estimated in the present study (72% and 22%, respectively).

Our study has some limitations, mainly related to the use of questionnaires. Self-reported tobacco consumption in cross-sectional studies is a reliable method of ascertaining smoking prevalence [24, 25], but we cannot exclude some information bias, which could have led to an underestimation of the prevalence of smoking [26–28]. However, previous studies have shown concordance and a high validity of self-reported smoking status when assessed against biological measures [29]. The exclusion of mobile-only telephone users could have resulted in a selection bias. Estimates indicate that 10.3% of Spanish households rely exclusively on cellular telephones. In addition, to avoid problems related to the length of the questionnaire, some variables, such as socio-economic or employment status, were not assessed.

Unfortunately, the possible role of the social crisis in the changes observed in smoking behavior is not easy to assess. Even the increase in the smokers smoking rolling tobacco may not be related to the crisis. However, the Spanish recession, which started in 2004, should be kept in mind as a possible confounder of the impact of the laws.

As policies are implemented, their effects vary over time. In this case, the study period was very short, and the law evaluation began a few months after its enactment.

A comprehensive law does not seem to be sufficient to significantly decrease the tobacco consumption in a population where the prevalence was already declining and specific actions need to be implemented in a stronger way [30].

Thus, the results of this study highlight the need to stimulate cessation, combining both a population strategy and an individual or high-risk strategy [31] through health promotion interventions prompting smokers to pass from the precontemplative stages to the contemplative stages (population strategy) and promoting specific tobacco cessation programs for those ready to quit (individual strategy).

## Supporting Information

S1 Dataset Supporting Dataset. (SAV)
